# Bioremediation of Textile Industrial Effluents Using Nutraceutical Industrial Spent: Laboratory-Scale Demonstration of Circular Economy

**DOI:** 10.3390/nano12101684

**Published:** 2022-05-15

**Authors:** Syed Noeman Taqui, Usman Taqui Syed, Raihan Taqui Syed, Mohammed Saeed Alqahtani, Mohamed Abbas, Akheel Ahmed Syed

**Affiliations:** 1 CSIR—Central Food Technological Research Institute, Mysore 570020, India; noemansyed89@gmail.com; 2 LAQV-REQUIMTE, Department of Chemistry, Faculty of Science and Technology, Universidade NOVA de Lisboa, 2829-516 Lisbon, Portugal; s.taqui@campus.fct.unl.pt; 3 Centre for Entrepreneurship and Business Incubation, Modern College of Business & Science, Muscat 133, Oman; syedrt85@gmail.com; 4 Radiological Sciences Department, College of Applied Medical Sciences, King Khalid University, Abha 61421, Saudi Arabia; mosalqhtani@kku.edu.sa; 5 BioImaging Unit, Space Research Centre, Michael Atiyah Building, University of Leicester, Leicester LE1 7RH, UK; 6 Electrical Engineering Department, College of Engineering, King Khalid University, Abha 61421, Saudi Arabia; mabas@kku.edu.sa; 7 Computers and Communications Department, College of Engineering, Delta University for Science and Technology, Gamasa 35712, Egypt; 8 Centre for Advanced Research and Innovation, Glocal University, Delhi-Yamunotri Marg, SH-7, Mirzapur Pole, Saharanpur 247121, India

**Keywords:** circular economy, bioremediation, textile industrial effluent, Nutraceutical Industrial Spent, composites, SME

## Abstract

This research reports the first-ever study on abundantly available, environmentally friendly, low-cost and ready-for-use Nutraceutical Industrial Cumin Seed Spent (NICUS) as an innovative adsorbent for bioremediation of a bisazo Acid Red 119 (AR119) dye, a probable mutagen from textile industrial effluents (TIEs). The experiment at the laboratory scale is designed to suit the concepts of sustainability and valorisation under the domain of circular economy. The experimental *q_e_* value obtained was 96.00 mg g^−1^. The optimised conditions of parameters are as follows: pH of 2; adsorption time, 210 min; adsorbent dosage, 0.300 g L^−1^; particle size, 175 µM; initial dye concentration, 950 mg L^−1^; orbital shaking, 165 rpm and temperature, 50 °C, producing an impressive value of 748 mg of dye adsorbing on 1 g of dry NICUS. The adsorption capacity of NICUS obtained from the quadratic model developed for process optimisation gave values of 748 mg g^−1^. As a prelude to commercialisation, five variables that affect the adsorption process were experimentally studied. For the feasibility and efficiency of the process, a two-level fractional factorial experimental design (FFED) was applied to identify variables that influence the adsorption capacity of NICUS. The identified variables were applied to scale experiments by three orders. Nine isotherm models were used to analyse the adsorption equilibrium data. The Vieth–Sladek adsorption isotherm model was found to be the best fit. The pseudo-second-order reaction was the appropriate mechanism for the overall rate of the adsorption process. Mechanistic studies related to mass transfer phenomena were more likely to be dominant over the diffusion process. Techniques such as SEM, FTIR and CHN analysis were used to characterise NICUS. The dye-adsorbed NICUS obtained as “sludge” was used as a reinforcing material for the fabrication of composites using plastic waste. The physicomechanical and chemical properties of thermoplastic and thermoset composite using dye-adsorbed NICUS were evaluated and compared with NICUS composites. Prospects of integrating Small and Medium Enterprises (SMEs) into the circular economy of Nutraceutical Industrial Spent (NIS) are discussed.

## 1. Introduction

The third decade of the third millennium is probably the most crucial decade for ensuring water security [1]. Safeguarding water security is one of seventeen sustainable development goals recognised by the United Nations [2]. The principle of sustainable development is to maximise economic growth by decoupling from water consumption and wastewater expulsion [3]. Thus, water constitutes a major apprehension, impairment and pathway for sustainable development of industries that generate effluents containing large amounts of toxic and hazardous substances. According to the Water Footprint Network, the dyeing of textiles consumes 9.00 trillion litres of water every year [4]. The massive amounts of water used in textiles have generated textile industries’ dubious distinction as one of the most pestilential industries in the world [5]. Moreover, the lack of stringent regulations in many developing countries means the textile producers dump wastewater directly into waterways, which are affecting the environment and the ecology very seriously [6]. Thus, sustainable index measurements of textile production assume paramount importance [6].

The sustainable index considers the impact on human health due to pollution arising from the production of textiles, placing emphasis on the management of the effluents. Such transitions toward sustainability have placed emphasis on the importance of water management and waste treatment in textile industries. The latter assumes importance as one of the key challenges of today’s economy, which is addressed through the concept of valorisation [7,8]. Valorisation is the process of conversion of waste into constituent parts for further utilisation, which has value beyond the cost of the process of transformation. It highlights practices and processes that decrease the discharges and reduces the environmental impacts. Unlike linear economics, which lay emphasis on end-of-life concepts [9], circular economy (CE) envisages the concept of reusing the waste of one process as a resource for another process [10]. Accordingly, both concepts attempt to decouple economic growth from natural resources through the process of reduction, reuse, recycling and returning to attain the status of a sustainable economic system [11]. Thus, valorisation and CE have been placed before the world to redesign laboratory research in tune with newer concepts.

Azo dyes extensively used in textile and allied industries comprise about 70% of the total synthetic organic compounds. These compounds are proscribed all over the world for carcinogenic and mutagenic properties. Despite their established harmfulness, they are extensively used in developing countries for their demonstrated advantages such as (i) simple and cost-effective methods of synthesis in aqueous media, (ii) the availability of a colossal choice of starting materials, (iii) a wide spectrum of shades, (iv) high intensity and superior fastness of the colour, (v) versatility in applications on a variety of substrates and (vi) the energy-saving dyeing process at 60 °C compared to the boiling temperature of its counterparts, which have made them important and indispensable to the textile industry [12]. However, due to the lack of suitable techniques to dispose of ~4.50 × 10^5^ tons of dyes per annum worldwide, these dyes are released as textile industrial effluents (TIEs) [13]. The Nutraceutical market is projected at US $336 billion by 2023 at the compound annual growth rate of 8% per annum [14]. However, the processing and production of principal components and/or active ingredients in the Nutraceutical market generates a voluminous 50–95% of spent. Though no figures are available about the total amount of the spent generated, considering the economics of the Nutraceutical market, it may run into millions of tons.

Cumin belongs to the family *Apiaceae*, which contains cyminum as a single species. The cumin seed has been known to humankind for centuries. Its total annual world production is estimated at 4.00 × 10^5^ MT [15]. The seeds have two components: the pleasant odour due to volatile oil content and cuminol, also named cuminaldehyde. The latter is the principal component that has medicinal value. The extraction of the principal component and oil content by mechanical, thermal and chemical processes generates a by-product commonly known as Nutraceutical Industrial Cumin Seed Spent (NICUS). NICUS has no feed or fertiliser value. Presently, the spent is used as a fuel with low calorific value due to its porous structure, which traps moisture and enhances carbon footprints.

A survey of the literature revealed that only one research article has reported on the decolourisation of AR119, a bisazo dye from textile industrial effluents [16]. The methods reported for azo dyes are broadly classified as biological [16], chemical [17] and physical methods [18,19]. Among these, biological methods fall short of achieving the degradation of AR119 dye, which is designed to remain a stable and long-lasting colourant and resist microbial degradation. The use of chemical treatment methods imposes a high operational cost of the treatment and disposal of large amounts of chemical sludge that contravenes the environmental sustainability and financial feasibility of industries [20].

Adsorption is a physical method for the remediation of AR119 using sewage sludge and sewage sludge ash. This method is user-friendly, easy to operate and incurs low maintenance costs [19]. The use of the aforesaid materials involves two pretreatments, namely, drying and incineration. The economic aspects of the drying process are greater compared to incineration [21]. However, the latter leaves a carbon footprint and involves the Environmental Impact Factor, also commonly known as E-factor [22]. These deficiencies have encouraged the authors to use NICUS as a superior and innovative adsorbent for bioremediation of AR119 from TIEs, which has the following characteristics: (i) it is ready-for-use adsorbent material for the removal of AR119 from TIEs by providing sustainable, clean technology, (ii) abundantly available, (iii) eco-friendly, (iv) low-cost, (v) provides a tenable solution to valorise Nutraceutical Industrial Spent (NIS), (vi) contains prospects to fabricate low-cost green composites using plastic waste and dye-adsorbed spent as filler material, (vii) has a competitive edge over reported agriculture waste and (viii) fosters innovation and suits moderate technology adopted in SMEs.

SMEs are the backbone of all economies in the world and are likely to play an important role in the success of the CE. They are a part of business and depend heavily on an innovation-friendly environment. There are 30 million SMEs in the USA alone [23], and they contribute to two-thirds of employment in Europe [24]. The WRAP and Green Alliance report on recycling and remanufacturing sectors mentions an estimated 205,000 jobs can be created in the United Kingdom by adopting the CE concept [25]. The Global Innovation Index 2020 is proactively boosting innovation-driven entrepreneurship and economic growth [26]. However, barriers such as lack of financial resources and lack of technical skills pose challenges in their transition to CE.

The present work focuses on the first-ever study on the use of NICUS for remediation of textile effluents by simple adsorption technology. Additionally, one of the objectives of the study is to replace charcoal that is extensively used in the industries. Charcoal as an adsorbent has serious limitations of high cost and high E-factor for the regeneration of the material [27]. One of the principal targets of this study is to use dye-adsorbed NICUS, a waste material, as filler/reinforcing material using plastic waste to fabricate composites. This will meet the demands for a sustainable solution for bioremediation of textile industrial effluents. Additionally, the proposed methodology offers a laboratory-scale demonstration of the concept of circular economy and opportunity for SMEs to integrate with the circular economy of Nutraceutical Industrial Spent (NIS).

## 2. Materials and Methods

### 2.1. Materials

**Figure 1:**
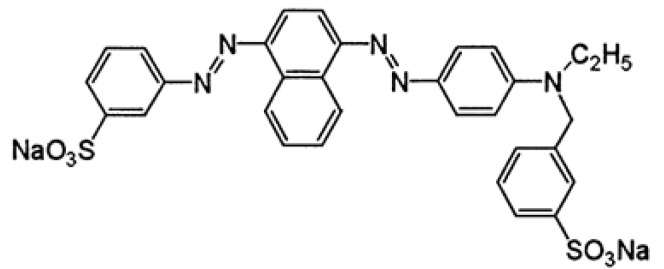
Structure of Acid Red 119 dye.

Acid Red 119 (AR119) dye was obtained from Sigma Aldrich, India. The dye is commonly referred to as Acid Red V (C.I. = 262,085; CAS registry number = 12220-20-1; chemical formula = C_31_H_25_N_5_Na_2_O_6_S_2_; molecular weight = 673.67. λ_max_ at 526 nm). A UV–vis spectrometer (Perkin-Elmer Lambda EZ-201, Waunakee, WI, USA) was used. The molecular structure of the AR119 dye is shown in Figure 1.

### 2.2. Studies on the Variables Affecting Adsorption of AR119 Dye on NICUS

The influence of variables affecting the adsorption of AR119 dye on NICUS was studied using batch experiments. The stock solution of AR119 (1000 mg L^−1^) was prepared using double-distilled water and was used to prepare the required 25–500 mg L^−1^ concentration solutions. To a series of 250 mL Erlenmeyer flasks, 50 mL aqueous AR119 dye solution (25–500 mg L^−1^) (adsorbate) was added. An amount of 50 mg of NICUS (adsorbent) was introduced into each Erlenmeyer flask. Three temperatures, 303, 313 and 323 K, were selected for the adsorption kinetic studies. Experiments were designed to study the effect of AR119 dye initial concentration (25–500 mg L^−1^) and dosage of NICUS (adsorbent) in the range of 0.025–0.300 g 50 mL^–1^/0.500–6.000 g L^−1^. The experiments were also designed to study the effect of pH 2, 4, 6, 7, 8, 10 and 12 on the process of adsorption. The pH of the solution was attuned using 0.01–1.00 M HCl or NaOH solution. All the trials were carried out using 200 mg L^−1^ as the initial dye concentration at almost neutral pH 7. During each trial, the solution was subjected to stirring in a thermostatic orbital shaker for 180 min at 165 rpm. Samples were withdrawn at predetermined equilibrium time. The unadsorbed AR119 dye in the solution phase was separated from NICUS by centrifugation at 3000 rpm for five minutes. If the solution was unclear, the centrifugation was repeated for an additional five min. The AR119 dye concentration at equilibrium pertaining to the supernatant centrifuged solution was determined using a UV–visible spectrophotometer. The coefficients of variance of the data obtained for the experiments carried out in triplicate did not exceed ±2% error.

### 2.3. Statistical Optimisation of Variables Affecting the Adsorption Process

The five independent variables affecting adsorption of AR119 dye on NICUS using batch experiments were time of contact of dye and adsorbent (A), the temperature of the system (B), initial dye concentration (C), adsorbent dosage (D) and pH (E) of the solution, using two-level Fractional Factorial Experimental Design (FFED) to optimise the adsorption capacity of NICUS statistically. The data were fitted to a second-order polynomial model for calculating the optimum conditions to obtain a quadratic regression equation. The empirical second-order polynomial model is as follows:

$$Y=\beta_{0}+\sum \beta_{i}X_{i}+\sum \beta_{ii}X^{2}+\sum \beta_{ij}X_{i}X_{j}.\;$$

where *Y* represents the dependent response variable, *β*_0_ is a regression coefficient, *β_i_* is the linear effect, *β_ii_* is the squared effect and *β_ij_* is the interaction effect of independent variable *X*. Statistical software was used for a Response Surface Methodology study and graphical representation of 3D and contour plots for the effect of independent variables on the response. Analysis of variance (ANOVA) was used as a tool for the analysis of data obtained from polynomial models using 95% confidence level and a value of the coefficient of determination *R*^2^ ≥ 0.90.

## 2.4. Characterisation Methods

IR spectra were recorded using the FTIR spectrophotometer (Perkin Elmer 3 lambda, USA). JEOL model 3300 (Japan) scanning electron microscope was used to record SEM images. A pH meter Model 802 from Systronics, India was used to measure pH.

### 2.5. Analysis of Adsorption Kinetics

The models provide an insight into the performance of adsorption of AR119 dye on NICUS with time as an independent variable. This is of significance to scale for commercial applications. To provide the variation in adsorption rate, a concentration of 100, 200 and 300 μg mL^−1^ of AR119 dye was used to carry out kinetic studies at 303, 313 and 323 K. The kinetic data of adsorption of AR119 on NICUS were analysed using pseudo-first-order Equation (1) and pseudo-second-order Equation (2) [28]. The results from each of the adsorption kinetics were statistically analysed. Based on one of the statistical parameters, coefficient of variance, *R*^2^, and the error function *χ*^2^ Chi-squared test, the model that best described the results of each experiment was determined out of the two evaluated kinetic models, namely, pseudo-first order and pseudo-second order. Subsequently, the constants of each equation and the equilibrium capacity (*q_e_*) were calculated. Table 1 shows the calculated values of the constants corresponding to each of the adsorption kinetics models and equilibrium times. The model that best described the data for all the experiments was the pseudo-second order.

*q* = *q*_*e*_ (1 − *e*^−*k*_1_*t*^) (1)

(2) $$q=\frac{q_{e}^{2}k_{t}t}{1+q_{e}k_{t}t}$$

Mathematical models of adsorption reaction and adsorption diffusion are proposed to determine the importance of diffusion in the adsorption process of the adsorbate onto adsorbent [29,30,31,32,33,34,35]. We have resorted to a functional empirical relationship of the uptake of the substrate at a given time *q_t_* varying almost proportionally with *t*^1/2^. This was performed by fitting an intraparticle diffusion model, Equation (4). These results demonstrate that the process of adsorption is not rate-limiting, and the progression of adsorption takes place in multiple steps. Thus, it may be envisaged that the movement of AR119 dye molecules onto the surface of NICUS proceeds to the diffusion into the pores of NICUS.

**Figure 2:**
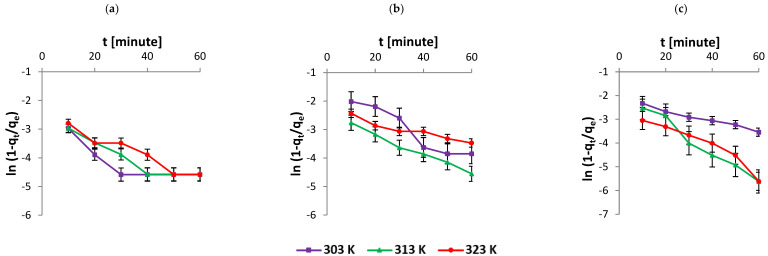
Kinetics data fitted to the film diffusion model with initial AR119 dye concentration (**a**) 100 µg mL^−1^, (**b**) 200 µg mL^−1^ and (**c**) 300 µg mL^−1^.

Analysis of adsorption kinetics data confirmed multiple levels of linearity, which in turn suggests multiple mechanisms. Higher concentrations and higher temperatures lead to higher adsorption rates, which lead to different linear routes. However, the process of adsorption becomes stabilised with respect to time. This was observed in a film diffusion model, Equation (3) [36]. It can be seen in Figure 2 and Table 2 that the values of diffusion constant *R*′ of a liquid film are in agreement with high *R*^2^ values. Furthermore, the values of *R*′ infer fast adsorption of a thin film onto the surface of the solute particles. The phenomena retard the process of diffusion, which affects the rate of adsorption. This step confirms that the adsorption process is limited by the diffusion phenomenon.

**Figure 3:**
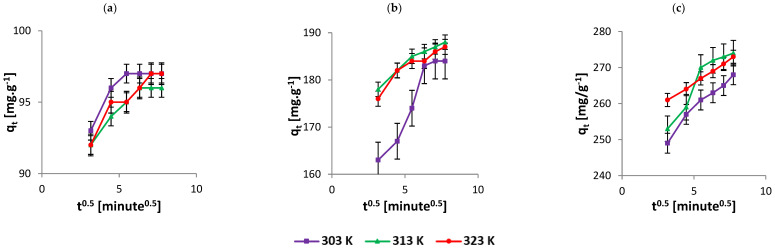
Kinetics data fitted to the Weber–Morris model with initial AR119 dye concentration (**a**) 100 µg mL^−1^, (**b**) 200 µg mL^−1^ and (**c**) 300 µg mL^−1^.

**Figure 4:**
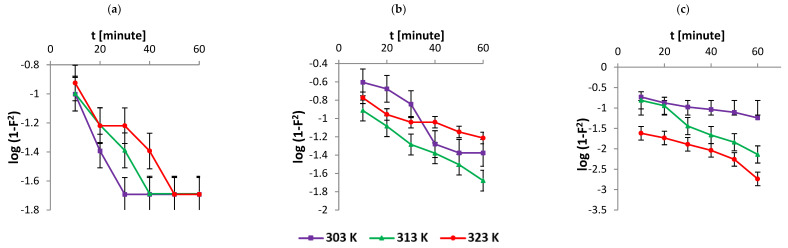
Kinetics data fitted to the Dumwald–Wagner model with initial AR119 concentration (**a**) 100 µg mL^−1^, (**b**) 200 µg mL^−1^ and (**c**) 300 µg mL^−1^.

According to the Weber–Morris model [37], the solute uptake varies with *t*^1/2^, as shown in Equation (5). A straight line was obtained on plotting qt versus *t*^1/2^. This observation conveys that intraparticle diffusion is the rate-limiting step for the process of adsorption of the dye on the adsorbent. Therefore, a straight line is anticipated for the plot qt versus *t*^1/2^ whose diffusion rate constant was obtained from the slope (kint) (Figure 3). Conversely, the Dumwald–Wagner model [38] calculates the true absorption rate (Equation (6)). The result infers the intraparticle diffusion as the rate-limiting step. The data are presented in Table 2 and Figure 4 [28].

ln(1 − *q_t_*/*q_e_*) = −*R*^1^*t* (3)

*q_t_* = *k_int_ t*^1/2^ (4)

*q_t_* = *k_id_t*^1/2^ + *C* (5)

*log* (1 − *F*^2^) = −*K*/2.303*t* (6)

**Figure 5:**
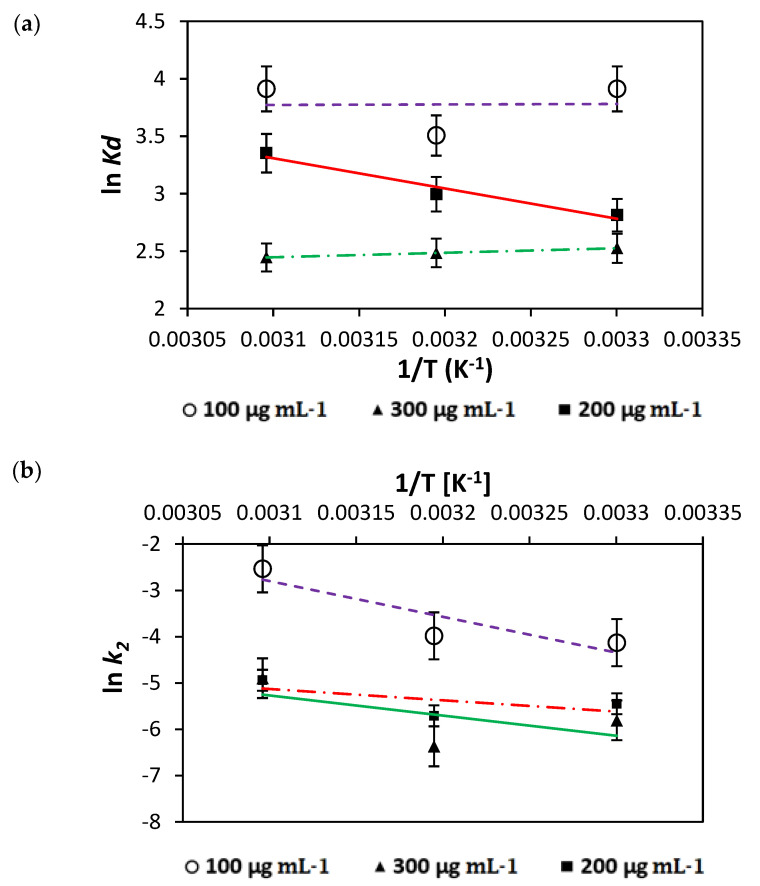
(**a**) Plot of the thermodynamic equilibrium constant versus 1/T to determine the enthalpy and Gibbs free energy of AR119–NICUS and (**b**) the pseudo-second-order kinetics for adsorption of AR119–NICUS.

The data obtained for the classical thermodynamic parameters, namely, ΔG°, ΔH° and ΔS°, indicate the nature and type of a reaction (Figure 5 and Table 3). For example, the positive ΔH° (enthalpy) values obtained from 303 to 323 K of NICUS indicate endothermic processes. The overall negative values of ΔG° (free energy) obtained for the AR119–NICUS system confirm the spontaneity and viability of the adsorption process. The magnitude of ΔG° values is indicative of rapid and almost spontaneous adsorption at lower temperatures. Further, it is inferred that the negative values of ΔS° (entropy) suggest minimum changes in the internal structure of the adsorbent and indicate the decrease in the randomness at the dye–NICUS interface.

### 2.6. Studies on Composites

#### 2.6.1. Preparation of AR119-Dye-Adsorbed NICUS

To a 100-litre barrel, 100 g of commercial AR119 dye was transferred. The dye was dissolved in 25 litres of TIE. An amount of 5 Kg of commercial NICUS was transferred, and the solution was stirred manually using a plastic rod that was 20 mm in diameter. The solution was kept for about 24 h with occasional stirring. The dye-adsorbed NICUS was separated using a cloth, and the precipitate was washed thoroughly with distilled water till the filtrate was almost colourless. The blue-coloured dye-adsorbed NICUS was air-dried. The resultant powder containing lumps was ground and sieved through 177 mµ mesh and dried in an oven at 60 °C for 24 h. The powder was cooled in a closed container with an airtight lid. AR119-dye-adsorbed NICUS was referred to as dye-modified NICUS powder (dm-NICUS).

#### 2.6.2. Preparation of the Composites

Thermoplastic bio-composites of polypropylene (polymer matrix) and NICUS (filler material) and dm-NICUS (filler material) were prepared as follows: polypropylene (PP) (H110MA) was purchased from Reliance, India (MFI = 11.0 g/10 min). NICUS and dm-NICUS were oven-dried at 100 °C for 12 h. Maleic anhydride-grafted-PP (MAg-PP) was used as a coupling agent. The thermoplastic composites of PP/dm-NICUS and PP/NICUS were prepared in three stages; first, dry-blending of PP resin with different proportions of 10, 20, 30, 40 and 50% (*w*/*w*) dm-NICUS and NICUS in a tumble mixer and melt compounding of master batches were conducted. The co-rotating fully intermeshing twin-screw extruder was used to mix the polymer matrix, coupling reagent and filler material screws and barrels. Third, the extrudates were collected, cooled and granulated into pellets. The injection-moulding process was used to prepare the dumb-bell specimens of the granulated blends and was tested for physicomechanical and chemical properties.

Thermoset composites of unsaturated polyester resin (USP) and dm-NICUS and NICUS in different proportions of 2, 5, 10, 15 and 20% (*w*/*w*) were prepared using 2% (*v*/*v*) methyl ethyl ketone as a catalyst. The following simple procedure was adopted. The mixture was agitated to obtain the homogenised slurry, which was transferred carefully to a glass frame of required dimensions. The slurry was allowed to dry. The resultant thermoset was placed under a pressure plate for about 3 h. A piece of required dimension as a sample was used to study the chemical and physicomechanical properties. Relevant ASTM procedures of ASTM D 570-98, ASTM D 638-95, ASTM D 792-00 and ASTM D 2240 were adopted to prepare thermoplastic and thermoset composites to study their properties.

## 3. Results and Discussion

### 3.1. Characterisation of NICUS and AR119–NICUS Surfaces

**Figure 6:**
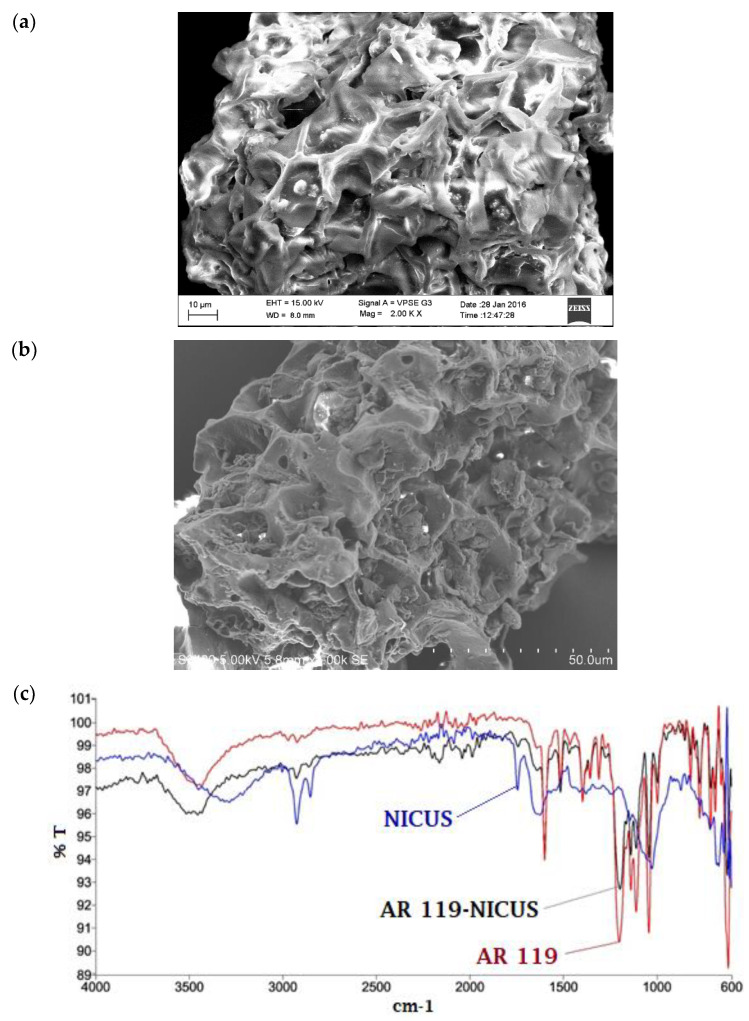
(**a**) SEM image of NICUS. (**b**) SEM image of AR119-NICUS. (**c**) FTIR spectra of AR119 dye, NICUS and AR119 dye adsorbed onto NICUS.

Surface characterisation of NICUS (Figure 6a) and dye-adsorbed NICUS (Figure 6b) was performed through SEM. The images display the surface of NICUS covered with the AR119 dye. The broadband near 3000–3500 cm^−1^ represents adsorbed water molecule and hydroxyl groups of cellulose (Figure 6c). An intense band at 3319 cm^−1^ supports –OH stretching. Additionally, it indicates possibilities of hydrogen bond formation to help in stabilising the conformations of certain macromolecules present in the adsorbent. Aliphatic C–H stretching bands appear at 2821 cm^−1^, 2904 cm^−1^ and 2923 cm^−1^, while C=O anti-symmetric stretching vibration of lignin is exhibited at 1521 cm^−1^ and 1775 cm^−1^. The phenolic, ester and ether groups are manifested as a doublet at 1390 cm^−1^ and 1310 cm^−1^, respectively. Additional bands at 1335, 1307, 1268, 1248 and 1016 cm^−1^ are ascribed to the C–O–C stretching.

### 3.2. Study of Independent Variables in the Adsorption Process

Keeping in view our design of the experiments targeted for commercialisation, all our studies were carried out at almost neutral pH 7.

#### 3.2.1. Effect of Time

**Figure 7:**
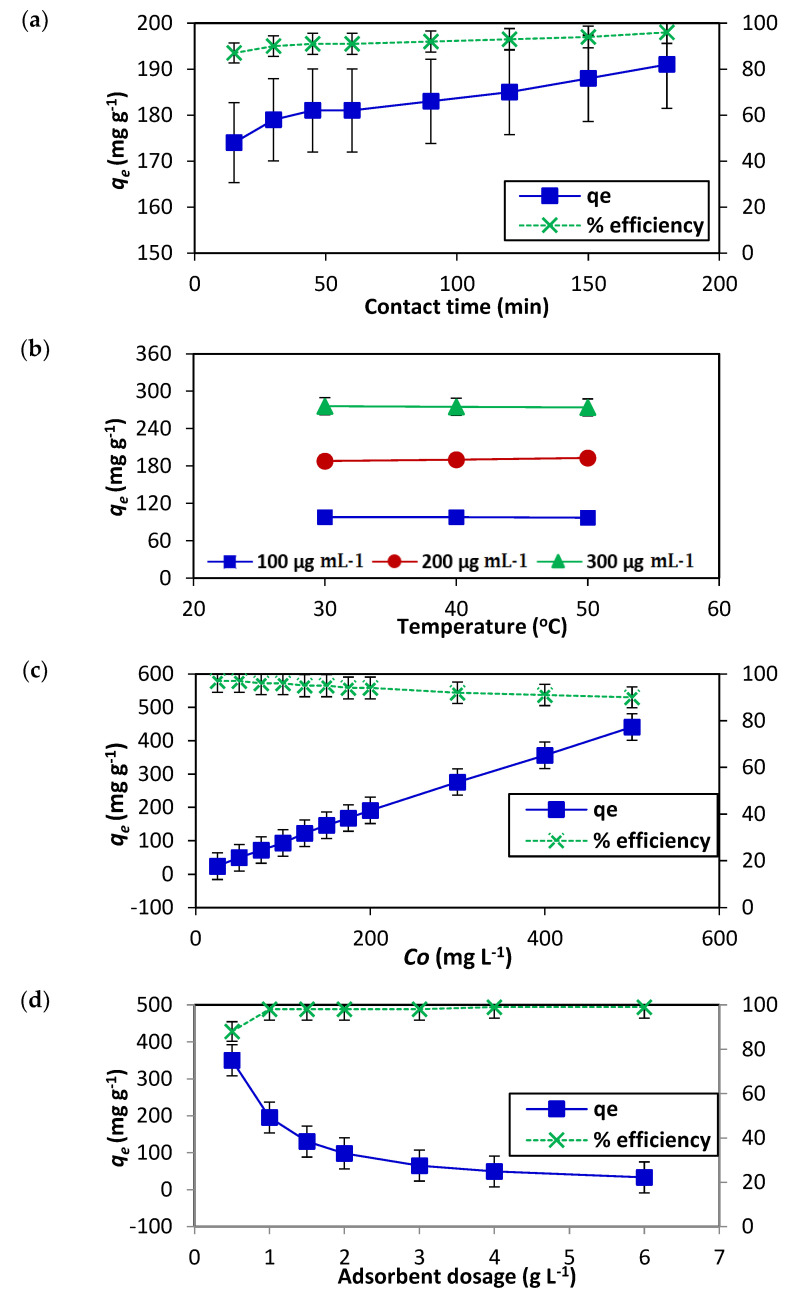

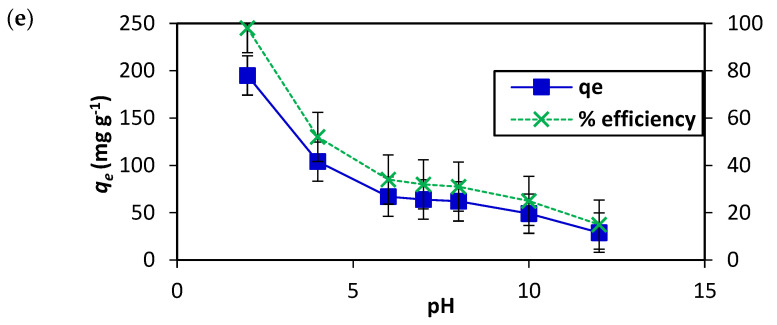
Effect of (**a**) contact time, (**b**) temperature, (**c**) initial dye concentration, (**d**) dosage and (**e**) pH on *q_e_* and percent removal efficiency of the AR119–NICUS system.

Adsorption as a separation technique is widely used for the elimination of toxic and hazardous substances from industrial effluents. The uptake of the dye by the adsorbent with respect to time at constant pressure and initial dye concentration helps to study adsorption kinetics by means of kinetic models. The influence of contact time on AR119 dye adsorption onto NICUS is presented in Figure 7a. From the results it can be concluded that the process of adsorption is almost spontaneous and efficiency of removal of the dye is about 90% within 15 min of the contact time. Thereafter, there is a marginal increase of about 7% in an additional 150 min.

#### 3.2.2. Effect of Temperature

Keeping in view our focus to scale to commercial applications, evaluation of the process of adsorption of dyes onto NICUS as a function of solution temperature was studied using Equations (7) and (8). The influence of temperature on AR119 dye adsorption onto NICUS is presented in Figure 7b. From the data presented, it is inferred that the process of adsorption is almost independent of the temperature range studied (30–50 °C).

ΔG° = ΔH° − ΔS°T(7)

ln *K_C_* = ΔS°/R − ΔH°/RT (8)

#### 3.2.3. Effect of Initial Dye Concentration

The adsorption capacity of NICUS is highly dependent on the initial AR119 dye concentration. This is manifested in the results displayed in Figure 7c. The shape of the curve suggests that the percent removal capacity of the adsorbate (AR119) by the absorbent (NICUS) is almost independent of the initial dye concentration in the range studied. This observation assumes paramount importance when the design is transformed to enhance the commercial viability of the technique.

#### 3.2.4. Effect of Adsorbent Dosage

The dosage as a parameter will also have a great influence on the commercialisation of the process because it decides the procedure’s economic feasibility. The range 0.50 to 6.00 g L^−1^ of the adsorbent dosage studied illustrated an influence of removal capacity of the dye only at lower concentrations (0.50 to 1.00 g L^−1^) and remains almost constant up until 6.00 g L^−1^. This observation shows that scientific and commercial importance is in increasing the number of trials with minimum amounts of the adsorbent, substantially increasing the dye’s removal efficiency by NICUS (Figure 7d).

#### 3.2.5. Effect of pH

The adsorption capacity of NICUS depends on solution pH. The pH plays two important roles; it influences, first, the characteristics of the adsorbent surface and, second, the chemistry of the dye solution [39]. The parameter, pH, is important to substantiate the efficiency of the adsorbent under study and plays a significant role to scale to commercial levels [30]. At lower pH, AR119, a bisazo dye, will be positively charged, which helps with the adsorption of NICUS, a cellulose material containing abundant –OH groups. As the pH increases, the positively charged dye gradually loses its positive character, which influences the decrease in the adsorption of the dye. The shape of the curve displayed in Figure 7e was consistent with the expected chemistry.

### 3.3. Adsorption Data Analysis Using Isotherm Models

The study of the isotherm models was intended to provide a view of the efficiency of NICUS for the remediation of the dye for commercial applications with an eye on the degree of economic advantages. The data of adsorption of AR119 dye onto NICUS were analysed using the adsorption isotherm models proposed by the Langmuir, Freundlich, Jovanovic, Toth, Brouers–Sotolongo, Sips, Vieth–Sladek, Radke–Prausnitz and Redlich–Peterson isotherm models. The main criterion of the study of adsorption isotherms was to select a model where *q_e_* (the experimental equilibrium) values were almost equal to *Q_m_* (monolayer adsorption capacity) values with a coefficient of variance (*R*^2^) value ≥ 0.90. To refine the results and to make a distinction between almost-similar data obtained by various isotherm models, *SSE* and *χ*^2^, as two additional error functions, were incorporated in our study.

**Figure 8:**
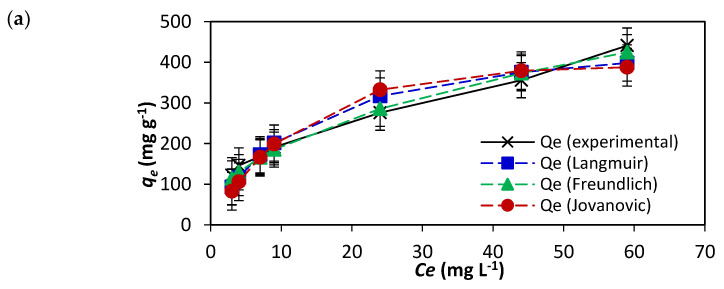

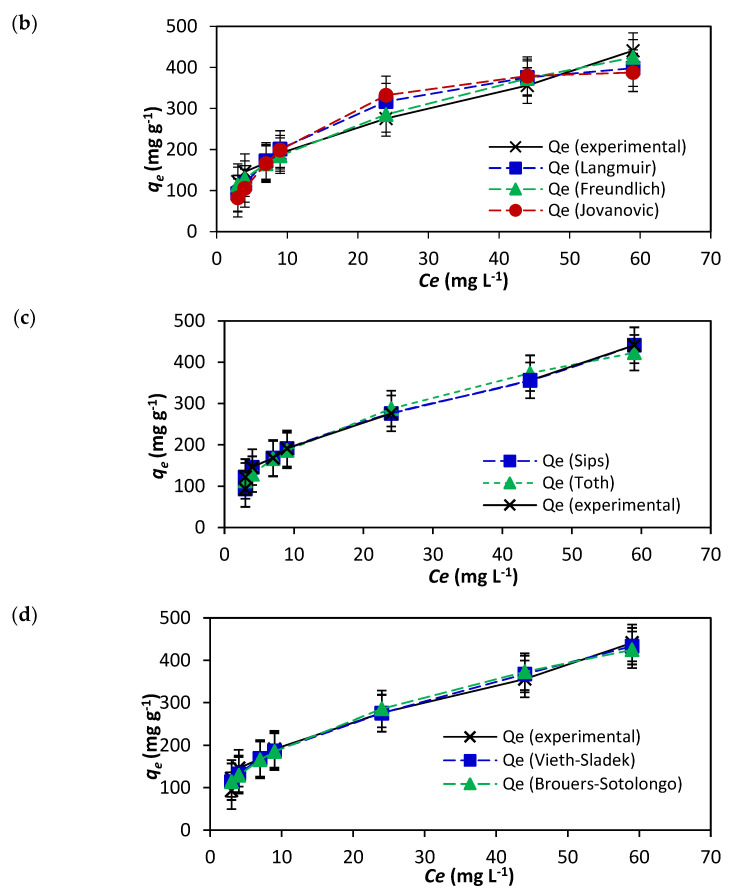
Fitting of adsorption data to (**a**) Langmuir, Freundlich and Jovanovic models, (**b**) Redlich–Peterson and Radke–Prausnitz models, (**c**) Sips and Toth models and (**d**) Vieth–Sladek and Brouers–Sotolongo adsorption isotherm models of the AR119–NICUS system.

Langmuir [40] proposed a model with the assumption that the adsorbent will have active sites possessing almost uniform energies. This was further established in the idea that no lateral interaction takes place between adsorbed molecules. A plateau in a two-dimensional graph with equilibrium concentration (*C_e_*) as an independent variable and *q_e_* (Figure 8a) as a dependent variable characterises the saturation of the active sites on the surface of the adsorbent. This implies that further adsorption cannot take place, and the possibility of multilayer adsorption of the dye is ruled out. The equations of the Langmuir isotherm model are shown in Equations (9) and (10). The experimental data, *R*^2^ = 0.95, *q_e_* = 96.00 mg g^−1^ and *Q_m_* = 483.40 mg g^−1^ and the separation factor (*R_L_*) values of 0.025 and 0.112 indicate adsorption of AR119 dye onto NICUS. If the increase in initial concentrations decreases the *R_L_* value, the adsorption process is considered more favourable. However, the variation between *Q_m_* and *q_e_* values of 483.40 and 96.00 mg g^−1^, respectively, for the AR119–NICUS system has provided impetus to explore other models. In contrast to the Langmuir isotherm model, Freundlich proposed heterogeneity of the surface sites with different energies of adsorption and demonstrated relevance to multilayer adsorption [41]. The mathematical expression is shown in Equation (11). The values of *n_F_* = 2.264 and 1/*n_F_* 0.442 (Table 4) of NICUS indicate that the process of adsorption is physical in nature and suits the Langmuir isotherm model’s behaviour.

(9) $$q_{e}=\frac{Q_{m}K_{a}C_{e}}{1+K_{a}C_{e}}$$

(10) $$R_{L}=\frac{1}{1+K_{a}C_{0}}\;$$

(11) qe=KFCe1nF

The Jovanovic model [42] attempts to minimise the deviancies of the experimental results obtained from the Langmuir isotherm model by introducing the exponential term *K_J_*. The mathematical model is presented in Equation (12). Upon comparing the difference in the values of *q_e_* = 96.00 mg g^−1^ and *Q_m_ =* 391.51 mg g^−1^ with the values obtained by the Langmuir isotherm model, one may surmise that values obtained with the Jovanovic isotherm model are better than that of the Langmuir isotherm model.

In the pursuit to identify a specific model(s) that has a smaller gap between experimental *q_e_* values and *Q_m_* values, six isotherm models, namely, Toth, Brouers–Sotolongo, Sips, Vieth–Sladek, Redlich–Peterson and Radke–Prausnitz, were also studied. The importance of these models is described elsewhere [43]. The mathematical Equation (13) represents the Radke–Prausnitz isotherm model [44]. The Redlich–Peterson isotherm model [45] in Equation (14) has a *‘g’* value of 0.559 as a correction exponent, which illustrates similarity to Langmuir isotherm model (Figure 8b). To describe a heterogeneous adsorption system, an empirical mathematical Equation (15) was developed by Toth [46]. The Sips isotherm model [47] (Equation (16)) combines the Langmuir and Freundlich adsorption isotherm models and suggests heterogeneity during the adsorption process (Figure 8c). Equations (17) and (18) represent the Brouers–Sotolongo isotherm model [48] and Vieth–Sladek isotherm model [49], respectively. The results are presented in Figure 8d.

(12) $$q_{e}=Q_{m}(1-e^{\left(K_{J}C_{e}\right)})$$

(13) $$q_{e}=\frac{K_{RP}Q_{m}C_{e}}{{\left(1+K_{RP}C_{e}\right)}^{m_{RP}}}$$

(14) $$q_{e}=\frac{A_{RP}C_{e}}{1+B_{RP}C_{e}^{g}}$$

(15) qe=QmCe(bTo+CenTo)−1nTo

(16) $$q_{e}=\frac{Q_{m}{\left(K_{s}C_{e}\right)}^{m_{S}}}{{\left(1+K_{S}C_{e}\right)}^{m_{S}}}$$

(17) $$q_{e}=Q_{m}[(1-\exp(-K_{BS}{\left(C_{e}\right)}^{\alpha })]$$

(18) $$q_{e}=K_{VS}C_{e}+\frac{Qm\beta_{VS}C_{e}}{1+\beta_{VS}C_{e}}$$

The study of nine isotherm models and evaluation of statistical parameters are presented in Table 5. In brief, the graphs obtained by nine models are similar in that they contain two parts, viz., a nonlinear part and a plateau. The former indicates that the dye molecule adheres to the active site of the porous NICUS, and the latter characterises the saturation of the adsorption process. Considering the values of *Q_m_*, *R*^2^, *SSE* and *χ*^2^, the Vieth–Sladek isotherm model fits best.

### 3.4. Statistical Process Optimisation using Two-Level FFED

Fractional Factorial Experimental Design (FFED) is a type of research method that allows the investigation of interaction effects between two or more variables. There are two types of variables, namely, independent variables and dependent variables. The value of the latter depends on the independent variables. These variables are also called factors. The factorial designs involve up to five factors. A two-level factorial design was investigated to evaluate the interaction effects of the factors run at two levels each, and only linear effects of the quantitative factors were studied (Table 6). The resultant data were improved and optimised using statistical and mathematical procedures using RSM. This methodology is extensively used in industrial processes to identify potentially influential parameters to redesign or improvise the production process. A two-level FFED is embedded in the central composite design (CCD). This design was adopted to study the quadratic effects of the factors suited to predictive modelling and optimisation. The former is a process that uses data and statistics to predict outcomes, while the latter is the action of making effective use of the data [50,51].

### 3.5. Quadratic Regression Equation

**Figure 9:**
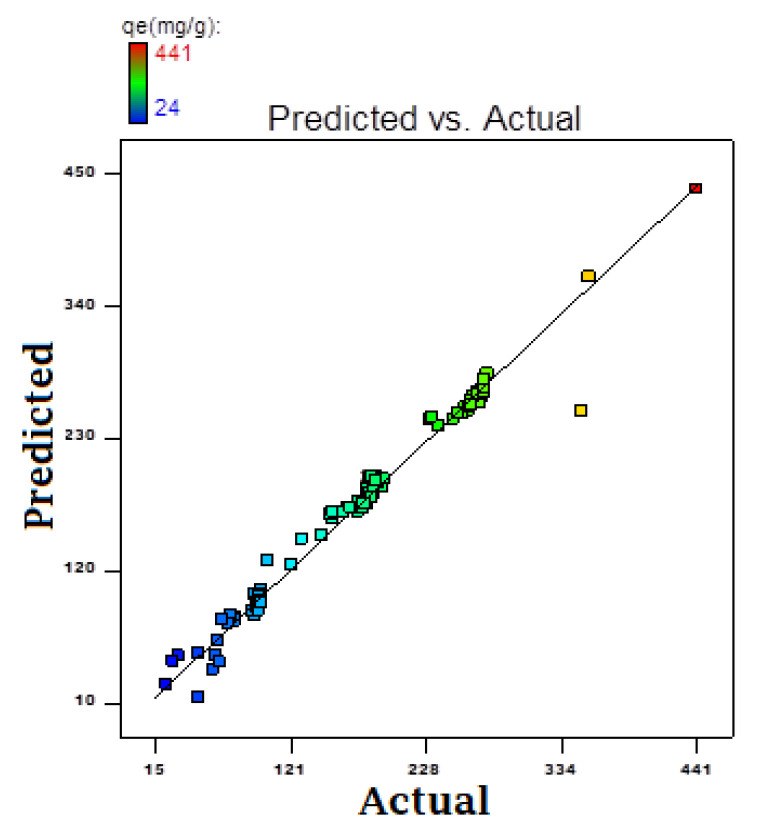
Actual versus predicted values of AR119–NICUS system.

The quadratic regression equation derived from analysis of variance (ANOVA) shows the possible individual and combined effect of the factors for the AR119–NICUS system (Table 7). It was observed that actual or experimental values are in conformity with the predicted responses (Figure 9). The regression equation for the AR119–NICUS system obtained is shown below:

$$AR119\;NICUS=-46.5+17.0\times A+0.9\times B+195.9\times C-102.1\times D-75.5\times E-3.5\times AB+9.3\times AC\;+2.3\times BC-16.0\times A^{2}-1.3\times B^{2}-20.2\times C^{2}+117.2\times D^{2}+58.3\times E^{2}\;$$

**Figure 10:**
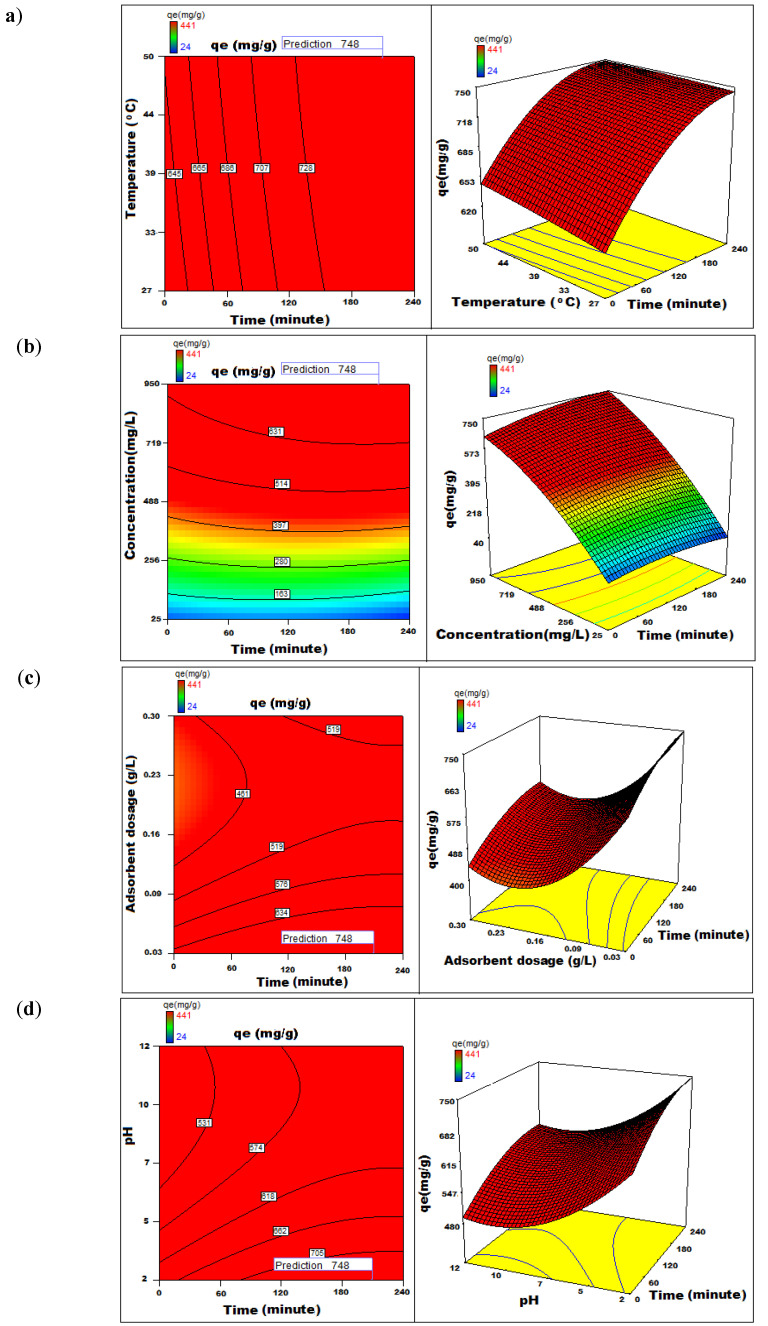
The 2D-contour and 3D-surface plots show the variation of adsorption capacity with (**a**) time versus temperature, (**b**) time versus concentration, (**c**) time versus adsorbent dosage and (**d**) time versus pH.

**Figure 11:**
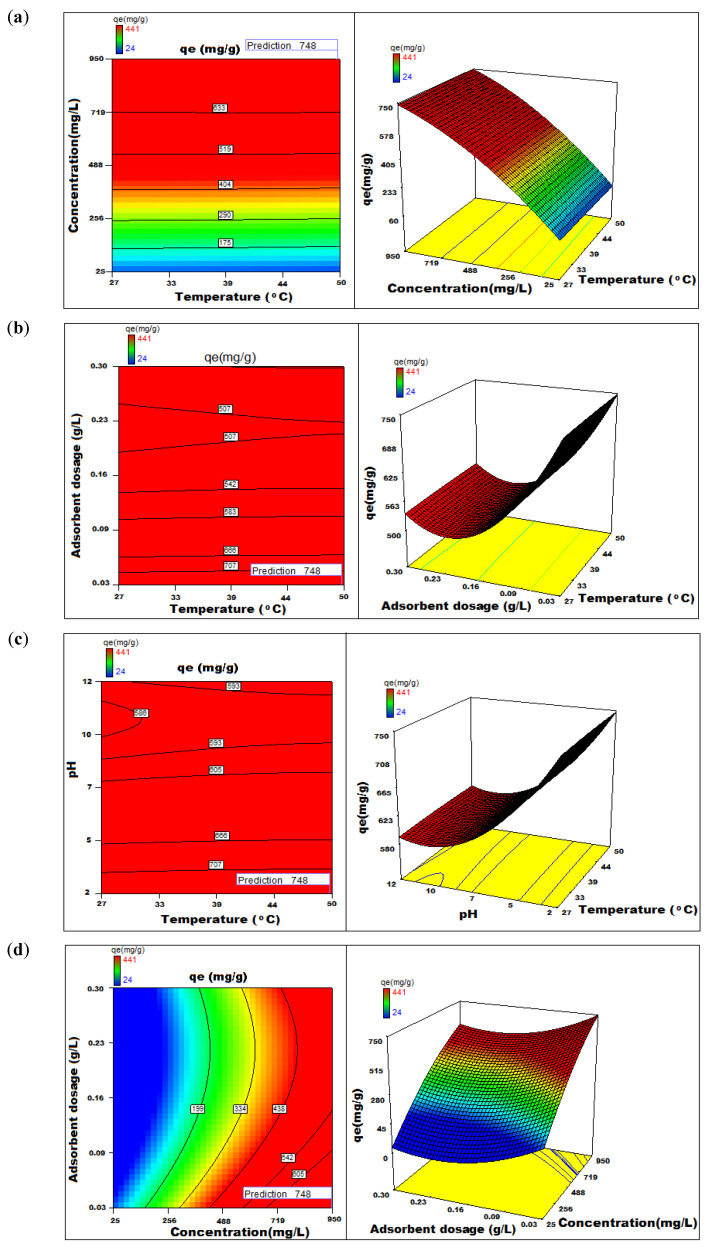

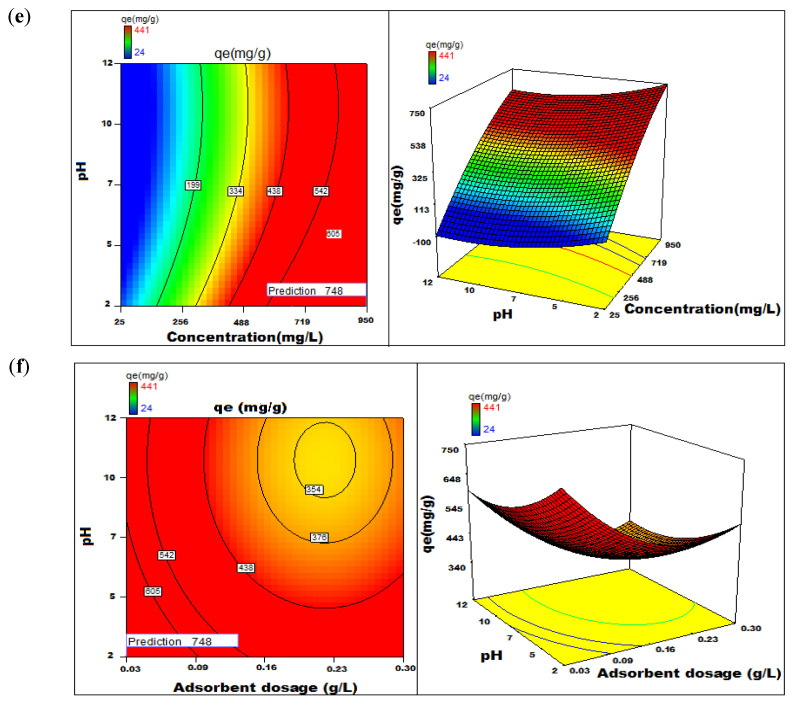
The 2D-contour and 3D-surface plots showing the variation of adsorption capacity with (**a**) temperature versus concentration, (**b**) temperature versus adsorbent dosage, (**c**) temperature versus pH, (**d**) concentration versus adsorbent dosage, (**e**) concentration versus pH and (**f**) adsorbent dosage versus pH.

The graph in Figure 10 and Figure 11 suggests a close interrelationship between the experimental data and expected responses. If the cross products of the variables, namely, A, B, C, D and E are zero, they are considered insignificant and not considered in the development of the regression equation. The multiple regression analysis based on CCD was obtained using the optimum values of variables studied using a second-order polynomial equation. The confidence interval of 95%, *p*-value < 0.05%, *F*-Value of 287, *R*^2^ value of 78.8% and adjusted *R*^2^ of 97.2 were considered imperative. The latter value of 97.2% and 7.9% coefficient of variance provides an opportunity to traverse into the systematic analysis and snip unwanted design points based on parameters of interest to indicate the effect of the variable(s) on the adsorption capacity. The contour and surface plots illustrate the combined effect of two factors on the process of adsorption, and the results are graphically presented in Figure 10 and Figure 11. In brief, the importance of statistical optimisation of the process variables leads us to interesting results which help to commercialise the process. At the optimised conditions of the parameters: pH 2; adsorption time, 210 min; adsorbent dosage, 0.300 g L^−1^; particle size, 175 µM; initial dye concentration, 950 mg L^−1^; orbital shaking, 165 rpm and temperature, 50 °C, an impressive value of 748 mg of dye adsorbs on 1 g of dry NICUS.

### 3.6. Analysis of 3D Response Surface Plots

The study on the effect of process variables and the data generated from the performed experiments showed an optimum time of 210 min for maximum adsorption at an initial dye concentration set at 200 µg mL^−1^. The temperature has only a marginally positive effect and increases along with time. This observation is helpful for the commercialisation process in the tropical countries where most of the Nutraceutical industries are located. The effect of initial dye concentration on the adsorption capacity of NICUS is marginal and remains almost constant in the range studied. A marginal decrease in the adsorption capacity was observed at higher dye concentration, probably due to expended active sites on the adsorbent. The adsorbent dosage has a negative effect on adsorption when plotted against time. However, the process of adsorption is improved by increasing the contact time, while pH has a negative effect with an increase in time on the adsorption capacity (Figure 10 and Figure 11). The maximum predicted adsorption capacity obtained through statistical optimisation was found to be 748 mg g^−1^.

### 3.7. Adsorption Process for Textile Industrial Effluents

**Figure 12:**
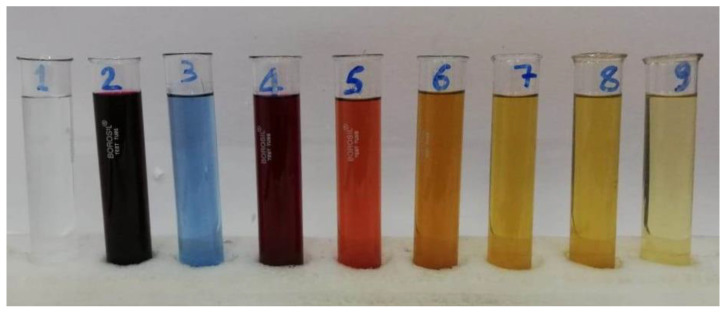
Colour of the solutions before and after bioremediation: 1. distilled water; 2. AR119 in distilled water; 3. TIE; 4. AR119 in TIE; 5. filtrate after adsorption of dye on NICUS after 15 min; 6. 30 min; 7. 45 min; 8. 60 min; 9. filtrate of NICUS in distilled water.

The details about the composition of the industrial textile effluent (TIE), sample collection and measurement of absorbance are adopted as detailed elsewhere [29]. Solutions of 1% (*w*/*v*) AR1190 were prepared by dissolving 5 g each of the dye in 5-litre distilled water (Solution 1) and in 5-litre TIE (Solution 2). Preliminary investigations were carried out to understand the factors responsible for the enhancement of the adsorption efficiency of the dye from aqueous matrices. We observed that the addition of fresh samples at short intervals provided better adsorption results. We also observed that the adsorbent had the capacity to eliminate the dye along with the allied materials present in Solution 2. The recovery of the dye by the process of adsorption measured from UV–visible spectroscopy was 98% (Figure 12).

To scale up the experiments by one and two orders, 0.5 and 5.0 g each of NICUS was transferred to 1-litre and 10-litre polyethylene beakers. The 500 mL and 5 L Solution 2 was added to 1-litre and 10-litre polyethylene beakers, respectively. Using a magnetic stirrer, the solutions were agitated. The procedure was repeated.

### 3.8. Characterisation of Composites

Physicomechanical properties of thermoplastic bio-composites of polypropylene and unsaturated polyester thermoset composites were studied. Additionally, the effect of ageing on tensile strength and the chemical resistance of unsaturated polyester thermoset composites was also evaluated. These results are detailed in Table 8, Table 9, Table 10 and Table 11.

A study of physicomechanical properties of thermoplastic PP composites suggests the increase in filler content of dm-NICUS or NICUS increased the tensile modulus and decreased elongation at break and tensile strength. Nevertheless, flexural properties improved substantially compared to neat PP. The presence of hydrophilic NICUS had an impact on the water adsorption properties, as manifested by the increase in weight shown in Table 8.

The results of our study suggest that thermoset composites of USP and dmNICUS/NICUS have better dimensional stability compared to USP. Moreover, our study also confirmed improved chemical resistance to all the chemical reagents studied except sodium hydroxide (10% *w*/*v*). However, with the increase in filler content, the tensile strength decreased probably due to reduced interfacial adhesion resulting in reduced interaction between the polymer matrix and the filler material.

### 3.9. Importance to Small and Medium Enterprises (SMEs)

A report on the global market of wood–plastic composites for the period 2020–2027 published by Grand View Research reported a value of USD 5.3 billion during 2019 and a projected CAGR of 11.4% for the period 2020–2027. The demand for wood–plastic composites is mainly due to better properties compared to conventional wood products, such as durability, shear strength, low moisture content and low water absorption. However, extensive research is occurring in order to replace wood with other cellulosic materials due to dwindling resources and stringent regulations against deforestation [52]. The use of bagasse, coir, corn Stover and stalks, jute, rice and wheat straw have been reported in the literature as a replacement for wood. However, the use of the aforesaid materials as fodder, fuel and feed restricts its use to the ever-increasing problem of the present century—food security, which also includes animal feeds. Thus, the use of Nutraceutical Industrial Spent (NIS), which has no feed, fertiliser and/or fuel value, embodies significant importance. Recently, the dye-adsorbed NIS as material in the fabrication of the composites using waste and/or virgin plastic has been reported [32].

The major barriers to the SME shift from a linear economy to CE include the following reasons: administrative burden, company’s environmental culture, innovation policies, technological know-how and privation of capital, effective legislation, government inadequate financial support and lack of information and technical back-up [53]. All these barriers are of no concern if the raw material is replaced with dye-adsorbed NIS or nanomaterials [54,55,56], which requires only optimising process parameters before production using the same machinery and know-how.

## 4. Conclusions

The adsorption capacity of NICUS obtained from the quadratic model developed for process optimisation produced values of 748 mg g^−1^ and followed the Vieth–Sladek isotherm model. Our adsorption of AR119 onto NICUS illustrated that the reaction is almost spontaneous in rate and endothermic due to low enthalpy values. Kinetic studies revealed the best fit matches with the pseudo-second-order model. Intraparticle diffusion was significant in mass transfer phenomena. The process is physical in nature, as evidenced by the low enthalpy values. SEM images and FTIR spectra confirmed the adsorption of the dye onto the adsorbent. In brief, stringent regulations, ever-increasing pollution due to textile industries and the high cost of activated charcoals have led to the resurgence of Nutraceutical Industrial Spent as a new class of low-cost, ready-for-use and abundantly available biomass, encompassing a better alternative to the available agriculture waste biosorbents. NICUS as an efficient adsorbent reduces grey water footprints, minimizes E-factor and maximizes water security of the textile industry. The use of dye-adsorbed NICUS “sludge” as a resource material for the fabrication of green composites using plastic waste reduces carbon footprints. What is more, the unattended challenge of the disposal of the sludge can be addressed to cater to the demands of a circular economy. In summary, an attempt has been made to provide an alternative paradigm using the sustainability concept in tune with the circular economy model. Dye-adsorbed NICUS as ‘waste’ generated after the remediation of the textile industry was used as a resource for composite industries using plastic waste. It is envisaged that the methodology, if adopted at a commercial scale, will have ample benefits in terms of economics and reducing carbon and water footprints by providing an alternative route for the problem associated with resource depletion—one of the major challenges of the 21st century. The authors hope that our endeavour will open up new routes to sustainability and green technology based on a circular economy to integrate SMEs.

## Figures and Tables

**Table 1:** Experimentally determined and theoretically predicted parameters for adsorption kinetics models.

Initial Concentration [μg mL^−1^]	Temp [K]	*q_e expt_* [mg g^−1^]	Pseudo-First Order				Pseudo-Second Order			
			*Q_m pred_* [mg g^−1^]	*k*_1_	*R* ^2^	*χ* ^2^	*Q_m pred_* [mg g^−1^]	*k*_2_	*R* ^2^	*χ* ^2^
**100**	303	98	96.05	3.15 × 10^−1^	0.91	0.04	97.78	1.61 × 10^−2^	0.95	0.01
	313	97	95.45	3.29 × 10^−1^	0.92	0.03	96.96	1.86 × 10^−2^	0.97	0.00
	323	98	97.60	5.08 × 10^−1^	0.90	0.01	98.01	7.91 × 10^−2^	0.90	0.01
**200**	303	188	179.26	2.27 × 10^−1^	0.91	1.14	188.14	3.00 × 10^−3^	0.91	0.42
	313	190	185.73	1.86 × 10^2^	0.92	0.10	65.88	1.71 × 10^−3^	0.90	1.65
	323	193	184.74	3.03 × 10^−1^	0.94	0.07	188.42	7.46 × 10^−3^	0.97	0.01
**300**	303	276	263.09	2.88 × 10^−1^	0.94	0.23	269.55	4.29 × 10^−3^	0.96	0.04
	313	275	270.13	2.68 × 10^−1^	0.92	48	278.41	3.31 × 10^−3^	0.91	0.12
	323	274	268.91	3.49 × 10^−1^	0.90	0.17	273.05	7.14 × 10^−3^	0.90	0.05

**Table 2:** Calculated parameters for diffusion models.

Initial Concentration	Temp	Film Diffusion Model		Weber–Morris Model		Dumwald–Wagner	
[μg mL^−1^]	[K]	*R*′ [min^−1^]	*R* ^2^	*k_ist_* [mg g^−1^ s^−0.5^]	*R* ^2^	*K* [min^−1^]	*R* ^2^
**100**	303	0.0289	0.90	0.79	0.90	0.029	0.90
	313	0.0344	0.91	0.89	0.91	0.034	0.92
	323	0.0362	0.93	1.04	0.93	0.036	0.93
**200**	303	0.0433	0.91	5.27	0.93	0.042	0.91
	313	0.0346	0.99	2.14	0.96	0.034	0.99
	323	0.0188	0.92	2.19	0.90	0.018	0.92
**300**	303	0.0255	0.98	3.90	0.97	0.022	0.98
	313	0.0634	0.98	4.87	0.90	0.063	0.98
	323	0.0479	0.92	2.62	0.99	0.048	0.92

**Table 3:** Experimental design of individual factors for RSM studies.

Factor	Name	Units	Minimum	Maximum
**A**	Time	minutes	0	180
**B**	Temperature	°C	27	50
**C**	Concentration	mg L^−1^	25	500
**D**	Adsorbent dosage	g L^−1^	0.500	6.000
**E**	pH		2	12

**Table 4:** Calculated parameters of adsorption isotherms.

**Two-Parameter Isotherms**											
**Langmuir**				**Freundlich**				**Jovanovic**			
*Q_m_*		483.4		*K_F_*		70.13		*Q_m_*		391.51	
*K_S_*		0.079		*n_F_*		2.264		*K_J_*		0.079	
**Three-Parameter Isotherms**											
**Toth**		**Brouers–Sotolongo**		**Sips**		**Vieth–Sladek**		**Radke–Prausnitz**		**Redlich–Peterson**	
*Q_m_*	4,237,909	*Q_m_*	2,034,296	*Q_m_*	17,050.7	*Q_m_*	194.3	*Q_m_*	4.2	*A_RP_*	23,457.4
*n*_*T*0_	0.059	*K_BS_*	3.45 × 10^−6^	*K_s_*	3.97 × 10^−6^	*K_VS_*	4.196	*k_rp_*	580,882	*B_RP_*	333,865
*b*_*T*0_	0.931	*α*	0.442	*m_s_*	0.442	β*_VS_*	0.367	*m_rp_*	0.558	*g*	0.559

**Table 5:** Statistical parameters for adsorption isotherm model fitting.

**Isotherms**	**Langmuir**	**Freundlich**	**Jovanovic**	**Toth**
**SSE**	5736.2	1467.3	9917.5	1560.5
*χ* ^2^	24.927	9.102	44.829	9.123
*R* ^2^	0.95	0.98	0.92	0.98
**Brouers–Sotolongo**	**Sips**	**Vieth–Sladek**	**Radke–Prausnitz**	**Redlich–Peterson**
1467.4	1467.6	914.9	1467.5	1468.1
9.102	9.102	7.297	9.100	9.099
0.99	0.99	0.99	0.99	0.99

**Table 6:** Thermodynamic parameters of AR119–NICUS system.

Initial Concentration	Temperature	ΔG°	ΔS°	ΔH°	ln A	E_a_
[μg mL^−1^]	[K]	[kJ mol^−1^]	[J mol^−1^ K^−1^]	[kJ mol^−1^]		[kJ mol^−1^]
100	303	−9.92	112.41	87.56	2.43	168.55
	313	−9.54				
	323	−9.15				
200	303	−7.89	185.92	158.37	8.21	300.69
	313	−7.69				
	323	−7.38				
300	303	−6.57	252.41	180.15	21.43	533.58
	313	−6.47				
	323	−6.36				

**Table 7:** ANOVA for fractional factorial experimental design of AR119–NICUS system.

Source	Sum of Squares	Degree of Freedom	Mean Square	F Value	*p* Value
**Model**	669,281.7	13	51,483.2	287.1	<0.001 **
**A**	6558.5	1	6558.5	36.6	<0.001 **
**B**	22.1	1	22.1	0.1	0.7264
**C**	255,634.1	1	255,634.1	1425.5	<0.001 **
**D**	42,967.9	1	42,967.9	239.6	<0.001 **
**E**	26,655.7	1	26,655.7	148.6	<0.001 **
**AB**	302.0	1	302.0	1.7	0.1977
**AC**	880.6	1	880.6	4.9	0.0292 *
**BC**	27.4	1	27.4	0.2	0.6966
**A^2^**	2294.8	1	2294.8	9.8	<0.001 **
**B^2^**	20.1	1	20.1	0.1	0.7386
**C^2^**	1068.4	1	1068.4	6.0	0.0166 **
**D^2^**	15,624.4	1	15,624.4	87.1	<0.001 **
**E^2^**	6005.7	1	6005.7	33.5	<0.001 **
**Residual**	16,318.4	91	179.3		
**Total**	685,600.1	104			

* Moderately significant (*p* value: 0.01 < *p* ≤ 0.05). ** Strongly significant (*p* value: *p* ≤ 0.01).

**Table 8:** Physicomechanical properties of thermoplastic polypropylene composites.

Properties	Percent Composition of Polymer Matrix and Filler Material											
	PP:NICUS						PP:dm-NICUS					
	100:00	90:10	80:20	70:30	60:40	50:50	100:00	90:10	80:20	70:30	60:40	50:50
**Tensile strength (MPa)**	30.8	29.6	28.6	26.8	24.4	18.7	30.8	29.8	28.7	26.4	23.3	19.6
**Tensile modulus (MPa)**	1040	1359	1548	1763	1727	1618	1040	1363	1538	1752	1745	1639
**Tensile elongation at break (%)**	156	13.2	10.2	6.2	3.8	3.1	156	13.4	10.6	5.9	3.9	2.9
**Flexural strength (MPa)**	33.2	49.9	52.3	54.1	55.7	NRR	33.2	51.3	53.1	55.6	56.1	NRR
**Flexural modulus (MPa)**	826	1497	1588	1753	2094	NRR	826	1130	1575	1787	2147	NRR
**Density (g.cm^−3^)**	0.904	0.928	0.988	1.014	1.059	NRR	0.904	0.928	0.986	1.015	1.063	NRR
**Surface hardness (shores D)**	70	74	76	79	84	NRR	70	74	75	78	86	NRR
**Water absorption in 48 h (%)**	0.01	0.14	0.28	0.80	2.67	NRR	0.01	0.15	0.30	0.81	2.87	NRR

NRR: non-reproducible results.

**Table 9:** Effect of ageing on tensile strength of unsaturated polyester thermoset composites.

Properties	Percentage Composition									
	USP:NICUS					USP:dm-NICUS				
	100:00	95:05	90:10	85:15	80:20	100:00	95:05	90:10	85:15	80:20
**Density (g mL^−1^)** **Experimental**	1.219	1.221	1.228	1.223	1.239	1.219	1.219	1.229	1.231	1.233
**Theoretical**	-	1.222	1.230	1.240	1.241	-	1.222	1.230	1.240	1.241
**Surface hardness** **(shores) ±2**	89.0	90.2	91.8	0.98	1.07	89.0	90.6	91.4	92.0	92.6
**Void content (%)**	-	0.47	0.89	0.95	1.04	-	0.45	0.86	0.96	1.11
**Specific tensile strength** **(KN m kg^−1^)**	37.0	25.6	25.1	21.2	19.1	37.0	25.8	25.3	21.3	18.9

**Table 10:** Physicomechanical properties of unsaturated polyester thermoset composites.

Properties	Percent Composition											
	USP:NICUS						USP:dm-NICUS					
	100:00	90:10	80:20	70:30	60:40	50:50	100:00	90:10	80:20	70:30	60:40	50:50
**Density (g cm^−3^) (experimental)**	1.219	1.221	1.228	1.223	1.239	1.219	1.219	1.229	1.231	1.233	1.219	1.221
**Density (g cm^−3^) (theoretical)**	-	1.222	1.230	1.240	1.241	-	1.222	1.230	1.240	1.241	-	1.222
**Surface hardness (Shores) (±2)**	89.0	90.2	91.8	0.98	1.07	89.0	90.6	91.4	92.0	92.6	89.0	90.2
**Void content (%)**	-	0.47	0.89	0.95	1.04	-	0.45	0.86	0.96	1.11	-	0.47
**Specific tensile strength (kN m kg^−1^)**	37.0	25.6	25.1	21.2	19.1	37.0	25.8	25.3	21.3	18.9	37.0	25.6

**Table 11:** Studies on chemical resistance of unsaturated polyester thermoset composites.

Chemical Reagents	Percentage Change in Weight After Seven Days								
	Neat USP	USP:NICUS				USP:dm-NICUS			
	100:00	95:05	90:10	85:15	80:20	95:05	90:10	85:15	80:20
**Water**	1.13	2.20	3.18	3.40	4.99	2.21	3.20	3.46	5.00
**10% (*v*/*v*) Acetic acid**	0.31	0.43	0.40	0.42	0.53	0.37	0.39	0.43	0.52
**10% (*v*/*v*) Hydrochloric acid**	0.36	0.41	0.50	0.52	0.56	0.40	0.48	0.51	0.53
**10% (*v*/*v*) Nitric acid**	0.40	0.45	0.64	0.70	0.72	0.44	0.62	0.68	0.70
**10% (*v*/*v*) Ammonium hypochlorite**	0.70	0.77	0.83	0.85	0.89	0.75	0.81	0.84	0.88
**10 % (*v*/*v*) Sodium hydroxide**	2.93	3.38	4.40	5.99	7.84	3.23	4.33	5.96	7.73

## Data Availability

Not applicable.
